# Ecological and genomic responses of soil microbiomes to high-severity wildfire: linking community assembly to functional potential

**DOI:** 10.1038/s41396-022-01232-9

**Published:** 2022-04-16

**Authors:** Nicholas C. Dove, Neslihan Taş, Stephen C. Hart

**Affiliations:** 1 Environmental Systems Graduate Group, University of California, Merced, CA USA; 2 Biosciences Division, Oak Ridge National Laboratory, Oak Ridge, TN USA; 3 Climate & Ecosystems Division, Lawrence Berkeley National Laboratory, Berkeley, CA USA; 4 Department of Life & Environmental Sciences and Sierra Nevada Research Institute, University of California, Merced, CA USA

**Keywords:** Microbial ecology, Biogeochemistry

## Abstract

Increasing wildfire severity, which is common throughout the western United States, can have deleterious effects on plant regeneration and large impacts on carbon (C) and nitrogen (N) cycling rates. Soil microbes are pivotal in facilitating these elemental cycles, so understanding the impact of increasing fire severity on soil microbial communities is critical. Here, we assess the long-term impact of high-severity fires on the soil microbiome. We find that high-severity wildfires result in a multi-decadal (>25 y) recovery of the soil microbiome mediated by concomitant differences in aboveground vegetation, soil chemistry, and microbial assembly processes. Our results depict a distinct taxonomic and functional successional pattern of increasing selection in post-fire soil microbial communities. Changes in microbiome composition corresponded with changes in microbial functional potential, specifically altered C metabolism and enhanced N cycling potential, which related to rates of potential decomposition and inorganic N availability, respectively. Based on metagenome-assembled genomes, we show that bacterial genomes enriched in our earliest site (4 y since fire) harbor distinct traits such as a robust stress response and a high potential to degrade pyrogenic, polyaromatic C that allow them to thrive in post-fire environments. Taken together, these results provide a biological basis for previously reported process rate measurements and explain the temporal dynamics of post-fire biogeochemistry, which ultimately constrains ecosystem recovery.

## Introduction

Wildfires are among the major processes shaping Earth’s ecosystems [1], and in many ecosystems, they are increasing in size, severity, and frequency, leading to fire regimes that are outside the historical range of variability [2]. Understanding how organisms recover from these severe disturbances is critical for predicting the states of ecosystems in the twentyfirst century and the numerous services they provide [3]. Soil microbial communities are particularly relevant in a post-fire ecosystem recovery context because they regulate essential nutrients, form symbioses with plants, and, in part, determine the fate of organic carbon (C)—all of which will influence plant regeneration and establishment [4]. Hence, the trajectory of ecosystem recovery from fire will be related to the taxonomic and functional succession of soil microorganisms.

Characterizing the microbial response to fire in a successional context recognizes that post-fire environments represent a gradient of recovery states. While previous studies have elucidated many factors that modulate the effect of fire on soil microbial communities such as burn severity [5, 6], soil depth [7, 8], and soil chemistry [5, 7, 9], the vast majority of these studies do not incorporate time since fire as a moderating effect even though such effects may last for decades [10]. Of those that evaluated soil microbial communities at multiple times since fire, it is clear that the post-fire soil microbial community is temporally dynamic [11, 12]. However, many studies are too short to capture microbial succession on time scales relevant to ecosystem recovery, especially in ecosystems with long-lived vegetation such as forests (i.e., decades). Given the constraints of long-term ecological research, it is not surprising that post-fire microbial succession is still unclear. However, space-for-time substitution approaches such as chronosequences have proven useful in understanding the long-term recovery of ecosystems to wildfire [13–17]. Studying long-term impacts of fire on microbial communities, and the successional nature of their assembly, would assist in predictions of how microbial communities will recover based on initial post-fire conditions.

Successional dynamics of microbial communities are also based on the relative balance of stochastic and deterministic processes governing microbial assembly [18, 19], which could influence the function of microbial communities [11]. However, it is unclear how the relative dominance of these ecological assembly factors may change with fire at time scales relevant to ecosystem recovery. In the near-term, 1–3 years after, fire may select for microbes that can withstand high heat [20], recolonize quickly after periods of dormancy [21–23], or capitalize on post-fire resources such as pyrogenic C (pyC) [24, 25]. Furthermore, altered soil chemistry directly after fire leads to environmental filtering resulting in highly selected communities [26]. Alternatively, in other successional contexts, the relative dominance of deterministic processes increases during ecosystem succession as competition among microbes for scarce resources grows [27, 28]. In longer-term, decade to century scales, the impact of wildfire on microbial assembly is likely multidimensional, and likely depends on the abundance and composition of recovering vegetation [29].

**Fig. 1:**
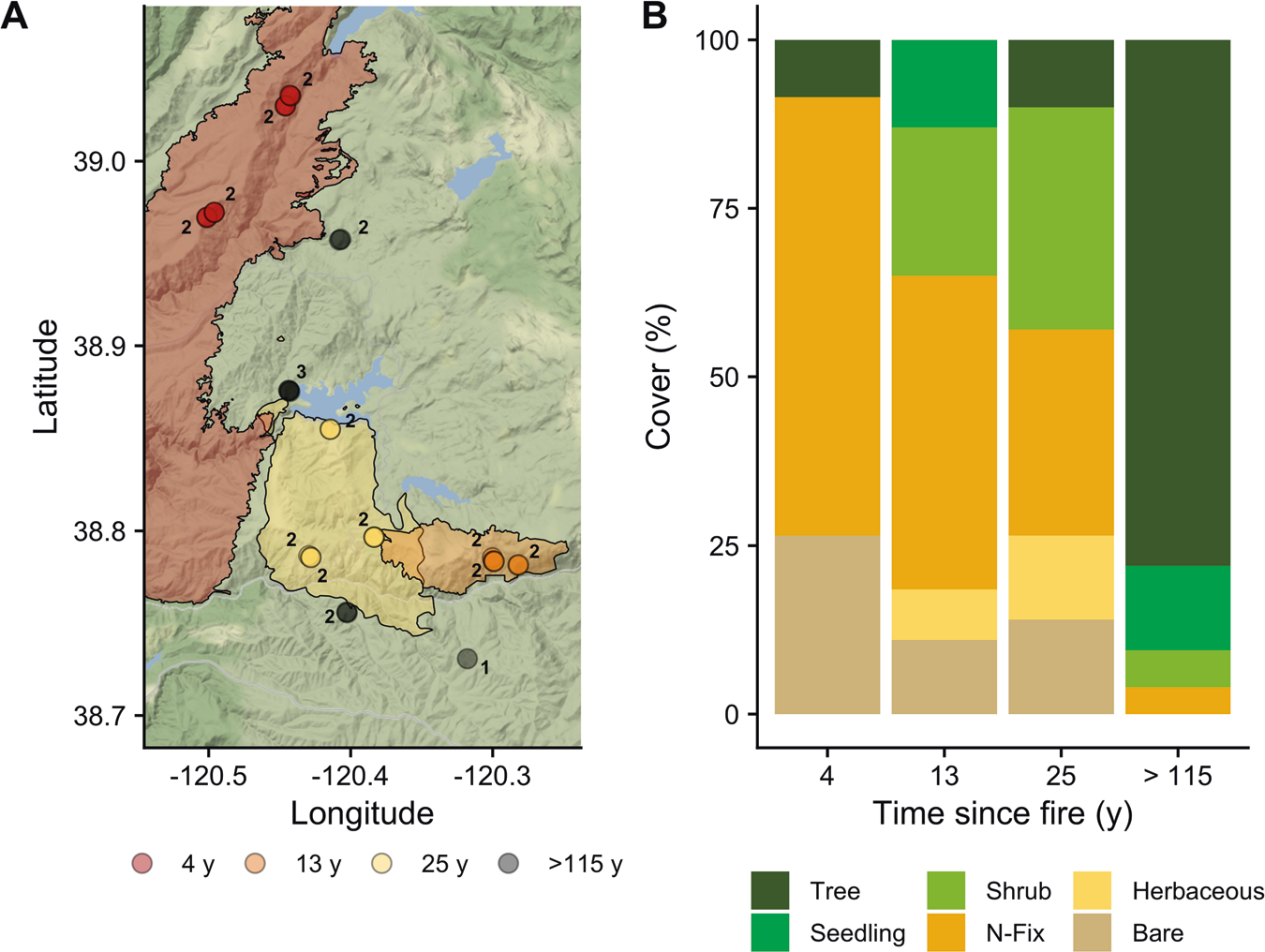
Map of the Central Sierra Nevada Fire Chronosequence (adapted from Dove et al. [13]). **A** Symbols denote approximate locations of plots and polygons show fire perimeters. Plots are at least 150 m apart and may not be visibly distinguishable at the spatial scale. Numbers next to points indicate how many plots are in the general location indicated by the points. **B** Mean percent plant cover types for each chronosequence site. Cover types are tree (*Abies concolor*, *Pinus ponderosa*, *Quercus* spp.), tree seedling (*Pinus ponderosa*), shrub (*Arctostaphylos* spp., *Salix* spp.), nitrogen-fixing plant (N-Fix; *Ceanothus* spp., *Chamaebatia foliolosa*), herbaceous (*Carex* spp., Poaceae), and bare soil.

Here, we employ high-throughput sequencing of the 16S rRNA gene for prokaryotes (archaea and bacteria) and the ITS region for fungi as well as shotgun metagenomics and reconstruction of microbial genomes (metagenome-assembled genomes [MAGs]). We pair these sequence data with previously published soil and vegetation data collected from the Central Sierra Nevada Fire Chronosequence [13] to elucidate long-term changes in soil microbial structure and function following high-severity wildfires (Fig. 1A). This chronosequence consists of sites that burned at high severity 4, 13, and 25 y prior to sampling in 2017, and they are compared to late-successional forests that have not burned within the past 115 y (see “Methods” for further details). Under this design, we address the following hypotheses: (1) the succession of microbial communities after high-severity fire follows distinct taxonomic and functional potential patterns resulting from differences in aboveground vegetation, soil chemistry, and microbial assembly processes; (2) ecosystem-level rates of potential decomposition and nitrate pools correlate with the genetic composition of microbial succession after fire; and (3) specific microorganisms thrive in discrete successional environments and harbor genetic traits that allow them to dominate at different post-fire successional states. We found that high-severity fire leads to long-term (>25 y) impacts to both microbial composition and functional potential that correlate with previously published rates of potential decomposition and nitrogen (N) pools [13]. Our results provide further evidence for the control of soil microbial communities over the bioavailability of soil N, potentially impacting the extent and rate of plant establishment, regeneration, and succession. Furthermore, while microbes 1 to 2 y after fire are characterized as fast-growing, ruderal species [30], we show that by year 4, certain microbes are highly adapted to post-fire conditions and possess the genetic capacity to degrade pyC. Our results underscore the importance of soil microbial communities in regulating post-fire ecosystem recovery and provide guidance in incorporating microbial ecology into post-fire forest management.

## Results and discussion

### High-severity fire results in a multi-decadal recovery of the soil microbiome, with concomitant differences in aboveground vegetation and soil chemistry

**Fig. 2:**
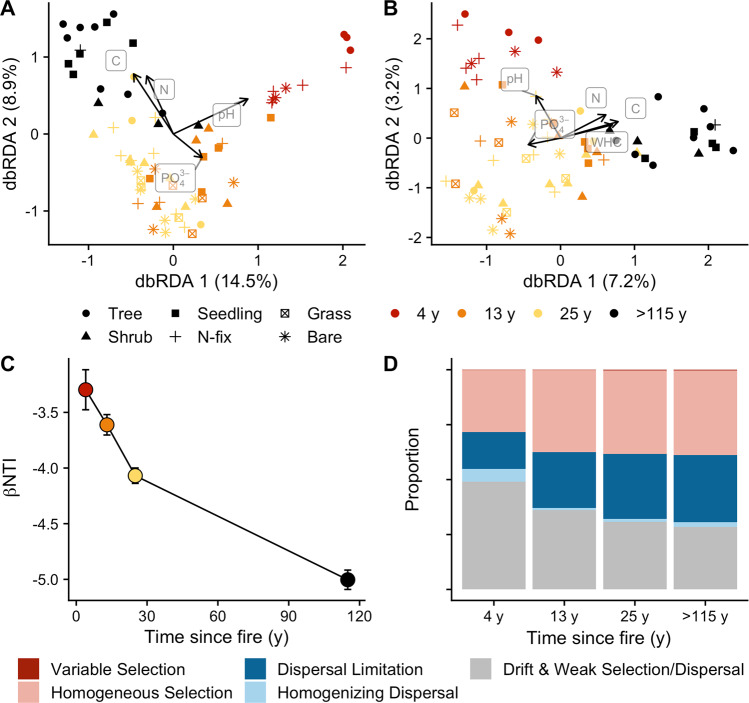
Controls on microbial community composition in the Sierra Nevada Fire Chronosequence. Distance-based redundancy analysis (dbRDA) ordination of prokaryote (**A**) and fungal (**B**) community composition along the variables: soil total carbon (C), total nitrogen (N), resin-available nitrate, resin-available ammonium, resin-available phosphate (PO_4_^3−^), soil pH (1:2 _w/v_ 0.01 M CaCl_2_), and water holding capacity (WHC). Only significant (*p* < 0.05) edaphic variables are plotted. Soil measurements are from Dove et al. [13] using the same samples used for molecular analyses. Points represent individual samples and are coded by time since fire and cover type based on color and shape, respectively. Vectors represent the direction and magnitude (indicated by vector length) of correlations of environmental variables with the first two axes of the dbRDA. Percentage in parentheses quantifies the variance explained by each axis. Note different axis scales. **C** Mean (and standard error, 4-y: *n* = 13, 13-y: *n* = 23, 25-y: *n* = 34, >115-y: *n* = 20) of whole-community prokaryote β-nearest taxon index (βNTI) within each time point, which quantifies the magnitude and direction of deviation between the observed null phylogenetic turnover distribution, plotted as a function of time since fire. Greater homogenous selection is indicated by decreasing βNTI. **D** The relative dominance of assembly processes calculated with the phylogenetic bin approach [37] for prokaryotes within each time point (variable selection is difficult to view at the scale of the figure).

Microbial community composition was significantly different among all post-fire stages (PerMANOVA – prokaryotes: *p* < 0.001, *R*^2^ = 0.34; fungi: *p* < 0.001, *R*^2^ = 0.18; Fig. 2, Fig. S1, Table S1). Even 25 y after fire, the microbial community composition was significantly different than the late-successional site (>115 y since fire, *p* < 0.001). When latitude and longitude were added to this model, only longitude was significant (prokaryotes: *p* = 0.008, fungi: *p* = 0.009), but the effect on the microbial community composition was negligible (*R*^2^ = 0.02 for both amplicons). As fire severity significantly moderates the microbial response [5], the multi-decadal differences observed in microbial community composition are tied to the severity of the fires studied here. For example, after 3 y since fire, soil microbial communities exposed to low-severity fire showed greater recovery compared to those affected by high-severity fire [10]. In some fire events, burn severities were low enough to not impact the microbial community at all [31]. Historical (pre Euro-American settlement) fires in this area were of low to mixed severity and occurred every one to two decades [32]. Our results show that the recovery of the microbial community to high-severity fire is longer than the historic fire return interval for this ecosystem and presumably longer than historical microbial recovery.

The relative dominance of different vegetative cover classes (e.g., tree, seedling, N-fixing plant, herbaceous, bare soil) among sites had a limited impact on changes in microbial community composition with time since fire (Fig. 1B). We found a significant but small vegetative cover type effect (PerMANOVA—prokaryotes: *p* = 0.019, *R*^2^ = 0.06; fungi: *p* = 0.016, *R*^2^ = 0.06, Fig. 2). We did not find significant interactions between the effect of time since fire and vegetative cover on microbial community composition (prokaryotes: *p* = 0.138; fungi: *p* = 0.563). However, significant differences emerged between grouping vegetation as trees/seedlings and herbaceous/bare (Table S2). Hence, the re-establishment of trees with time since fire appears to be related, in part, to microbial recovery. This was exemplified by shifting dominance of different fungal guilds. For instance, the relative abundance and richness of operational taxonomic units (OTUs) assigned to arbuscular mycorrhizal taxa were significantly greater under herbaceous cover types (ANOVA—relative abundance: *p* < 0.001, richness: *p* = 0.016, Fig. S2A, B). Furthermore, while the relative abundance of reads assigned to ectomycorrhizal (EM) taxa did not differ among cover types (*p* = 0.256, Fig. S2C), the richness of EM OTUs did (*p* = 0.017, Fig. S2D), where the highest richness was observed under tree seedlings. The relative abundance and richness of reads assigned to EM taxa increased logarithmically with time since fire (relative abundance: *p* = 0.003, rho = 0.32, richness: *p* < 0.001, rho = 0.67), recovering to that of the late successional site at 13 y since fire (Fig. S3). Given that the vegetation over the chronosequence shifted from herbaceous and N-fixing shrubs (e.g., *Ceanothus* spp.), which form symbioses with AM fungi, to trees (e.g., *Quercus* and *Pinus*), which form symbioses with EM fungi, changes in mycorrhizal dominance likely reflect post-fire vegetation succession. Relationships between the plant and microbial communities likely also emerge from the substrate quantity/quality and the physical conditions (e.g., degree of insolation and soil moisture content) found under different vegetative cover types that select certain microbes [33, 34]. Indeed, the establishment of plants early in post-fire succession correlates with microbial community composition and function [35]. However, we build upon this previous work to show that the post-fire succession of both above- and below-ground biota is linked decades after disturbance, particularly for mycorrhizal fungi and their respective hosts.

Differences in soil chemistry with time since fire were also significant in explaining the changes in microbial community composition (distance-based redundancy analysis—prokaryotes: 30%, *p* < 0.001; fungi: 18%, *p* < 0.001; Fig. 2). Overall, soil pH (which decreased with time since fire) was the strongest predictor for prokaryote community composition, and soil organic carbon (SOC; which increased with time since fire) was the strongest predictor for fungal community composition (Table S3). It is difficult to infer directionality in correlations between the microbial community composition and soil chemistry because microbes both control many biogeochemical cycling processes (e.g., C and N cycles) and respond to differences in substrate availability (e.g., C and N). However, many post-fire biogeochemical changes are abiotically derived, such as combustion of SOC, release of inorganic N, and increases in pH [36]. Under high-severity fires, changes in soil chemistry can continue for decades [13] and affect long-term microbial succession.

### Adaptation is key: selection increases with time since fire

To determine the impact of ecological assembly processes structuring soil prokaryotic communities in this chronosequence, we used two null modeling approaches. These quantified the relative dominance of deterministic (i.e., selective) and stochastic assembly for the whole community [18] and for individual groups of related taxa (i.e., phylogenetic bins) [37]. The first approach was used to extend modeling results from Ferrenberg et al. [12] and Knelman et al. [11], which evaluated the effects of wildfire on microbial community assembly within one year and after three years, respectively, to our longer-term chronosequence using the same null modeling framework [18]. The second approach uses a similar framework to Stegen et al. [18], but has been developed further to calculate the dominance of different assembly processes on phylogenetic bins of related taxa that may be dictated by factors different from those imposed on the whole community [37]. We excluded fungi from this analysis because the ITS gene region does not align well across large phylogenetic distances, precluding the use of ITS-based phylogenetic trees that are necessary for this analysis.

With the first approach (i.e., the whole community approach), we found that deterministic homogenous selection, consistent selective pressure homogenizing the microbial community propagated by environmental conditions, was the major driver of prokaryote assembly (73–99% range, Fig. S4A). The relative dominance of homogeneous selection (βNTI < −2) within each time point increased steadily during recovery (Fig. 2C). Prior work using the same modeling framework has demonstrated that, 4 to 16 weeks after wildfire, selective niche-based assembly processes are initially relaxed but then grow in dominance. Furthermore, 3 y into recovery, 89% of pairwise comparisons between communities were characterized by homogeneous selection [11, 12]. We build upon this previous work by showing that, over longer time scales relevant to forest recovery, the influence of selection grows even stronger with time since fire (Fig. 2C). Increasing selection is important because this suggests that post-fire prokaryote assembly and succession can be predicted based on ecological conditions and the genetic traits of the microbial populations.

Increasing homogeneity of the environment could lead to a dominance of homogeneous selection. However, unlike previous studies [38, 39], we did not find greater heterogeneity of soil chemistry in burned sites. For instance, we found that the coefficient of variation in SOC and pH (the two most important soil variables explaining microbial community composition) was similar with time since fire (Table S4). Instead, it is likely that pH exerted a selective pressure on the microbial community as evidenced by a significant positive correlation between βNTI and pairwise differences in soil pH (Mantel Test: *p* < 0.001, rho = 0.191; Fig. S4B). There was not a positive correlation between βNTI and pairwise differences in SOC (Mantel Test: *p* = 0.999; Fig. S4C).

The findings from the whole-community approach were largely supported by the phylogenetic bin approach, with increasing homogeneous selection within each time point over time (Fig. 2D). However, the phylogenetic bin approach also detected a significant proportion of stochastic assembly factors, such as drift, in the 4-y site that decreased with time since fire (Fig. 2D). In the 4-y site, drift was the primary assembly process for 136 out of 283 phylogentic bins, especially those bins dominated by Delta- and Gammaproteobacteria as well as the Cyanobacteria order Vampirovibrionales. It is possible that the wildfire reduced microbial abundances such that neutral “priority effects” increased in importance. Indeed, microbial biomass C increased with time since fire at these same sites [13], suggesting that wildfire had reduced microbial biomass and that the effect was long-lived. Hence, the acute disturbance of high-severity wildfire represents an important, chronic selective force affecting the microbial community composition and assembly processes decades after disturbance.

### Multi-decadal changes in microbial C metabolism post-fire correlates with rates of potential decomposition

**Fig. 3:**
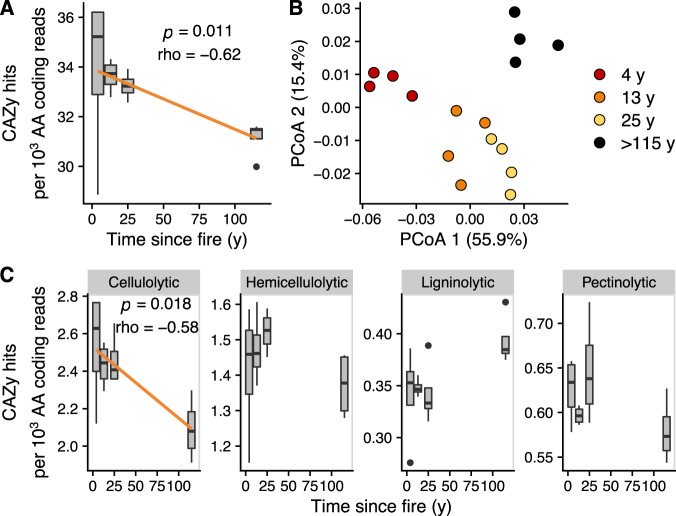
Distribution and composition of carbohydrate-active enzymes (CAZy) genes across the fire chronosequence. **A** Total abundance of CAZy genes, corrected by amino acid (AA) coding reads, are represented by boxplots, as a function of time since fire. **B** Differences in the composition of CAZy genes are represented using Principal Coordinates Analysis (PCoA) on proportionally normalized data with Bray-Curtis dissimilarities, and percentage in parentheses quantifies the variance explained by each axis. **C** Boxplots representing the normalized abundance of functionally classified CAZy genes as a function of time since fire. The orange lines represent the best-fit linear regressions where significant (Spearman correlation: *p* < 0.05, *n* = 4) relationships occur. Note different Y-axis scales.

Within the three decades after fire, microbial communities were significantly enriched in genes required for C metabolism compared their late-successional counterparts. The relative abundance of genes encoding for carbohydrate-active enzymes (CAZy) [40, 41] decreased with time since fire (*p* = 0.011, rho = −0.618, Fig. 3A). This was particularly true for the relative abundance of glycoside hydrolases (GHs), which reached a maximum at the 4-y site and, on average, decreased 14% from the 4-y to >115-y site (*p* = 0.009, rho = −0.631, Fig. S5). High relative abundance of GH genes could be indicative of low C availability because it represents investment in C acquisition [42]. For example, in a root mesocosm experiment, GHs were enriched in microbial genomes found in the relatively C-poor bulk soil compared to microbial genomes found in the relatively C-rich rhizosphere soil [43]. In another post-fire forest, there was an increased investment in C-acquiring extracellular enzymes 3 y after fire [11]. Taken together, these results suggest that microbial investment in C acquisition (possibly at the expense of efficient growth—e.g., [44, 45]) is relatively high after fire and remains elevated for decades (Fig. 3A). Relatively large investments in C acquisition is possibly a fire-adapted trait, because under severe fires, easily degradable SOC is depleted [13], leading to C-limited conditions. However, elucidating C limitation from CAZy abundances should be interpreted with caution, and future work should pair these data with transcriptomic and physiological measurements.

Substrate preferences of the microbial community also changed during post-fire succession, indicated by the shifting composition of CAZy genes with time since fire (PerMANOVA: *p* < 0.001, *R*^2^ = 0.69, Fig. 3B). This suggests that microbial communities not only change taxonomically, but also metabolically, during post-fire ecosystem recovery. To determine how microbial C metabolism shifts post-fire, we also classified a subset of gene families into functional groups based on C substrates [46–48]. The change in C metabolism was characterized by decreasing cellulose degradation with time since fire (*p* = 0.018, rho = 0.58, Fig. 3C). Changes in metabolic capacities are tied, in part, to changes in substrate and nutrient availabilities. For example, we also found that the richness of GHs increased with time since fire (*p* = 0.030, rho = 0.54), suggesting the diversity of C sources increased during post-fire recovery. However, greater cellulose degradation potential directly after fire may not be the result of increased cellulosic substrates, as they typically decrease after fire [49, 50]. Instead, greater cellulose degradation potential is a likely outcome of a C acquisition strategy in low C, post-fire environments. Cellulose has a greater substrate use efficiency than more complex C forms, such as lignin [51], so the production of cellulosic enzymes should be favored over ligninolytic ones when C is limiting.

**Fig. 4:**
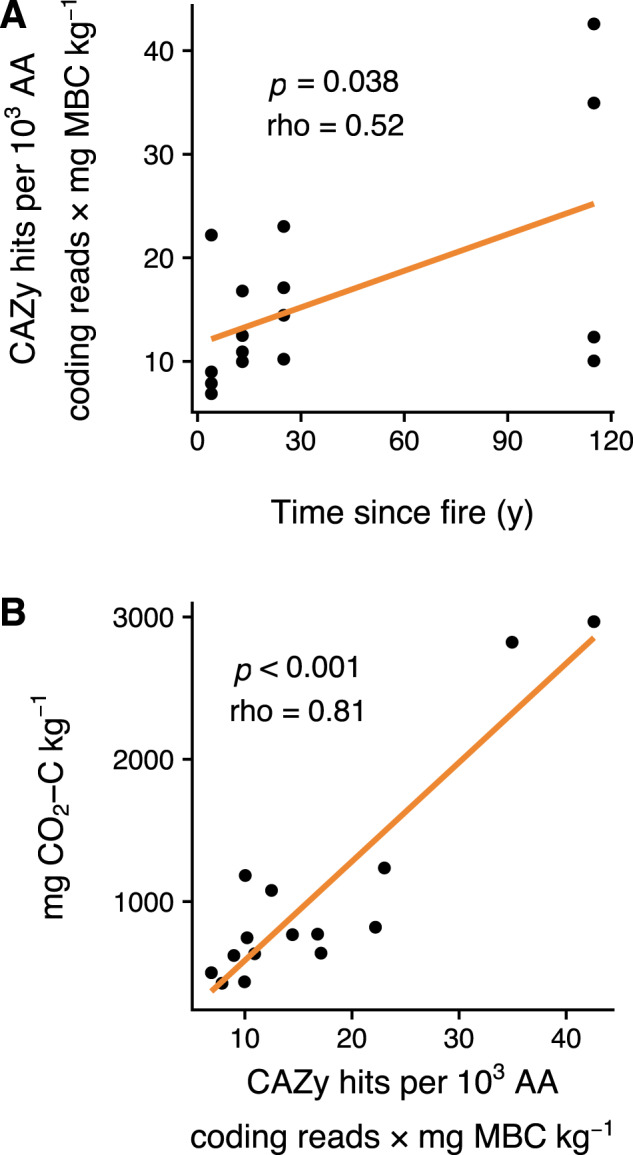
Genetic capacity for carbon degradation correlates with carbon dioxide (CO_2_) emissions. **A** Correlation between total abundance of carbohydrate-active enzymes (CAZy) genes, corrected by amino acid (AA) coding reads and multiplied by microbial biomass (MBC) with time since fire. **B** Correlation between cumulative CO_2_ emissions during a 28-day laboratory incubation with normalized CAZy abundance. The orange lines represent the best-fit linear regressions where significant (Spearman correlation: *p* < 0.05, *n* = 4) relationships occur.

To understand how CAZy genes affected ecosystem processes, it is necessary to benchmark these CAZy relative abundances to the size of the microbial community. We, therefore, multiplied our CAZy relative abundances by the microbial biomass of the same samples from Dove et al. [13]. Unlike the CAZy relative abundances, we found that these normalized CAZy abundances increased with time since fire (*p* = 0.038, rho = 0.52, Fig. 4A). Furthermore, we found that these normalized CAZy abundances positively correlated with cumulative CO_2_ efflux during a 28-d laboratory incubation of the same samples from Dove et al. [13] (*p* < 0.001, rho = 0.81, Fig. 4B). This suggests that increasing microbial biomass with time since fire outweighs the greater CAZy relative abundances of early successional microbial communities in determining potential rates of decomposition in post-fire soils. However, we also show that CAZy relative abundances, along with microbial biomass measurements, can be used to predict potential microbial decomposition. There is recent interest in modeling SOC re-accumulation after fire [52, 53]. Our results suggest that these models could be improved by incorporating microbial biomass and, potentially, microbial genetics.

### Differences in microbial N cycling capacity reflect inorganic N availability in post-fire soils

**Fig. 5:**
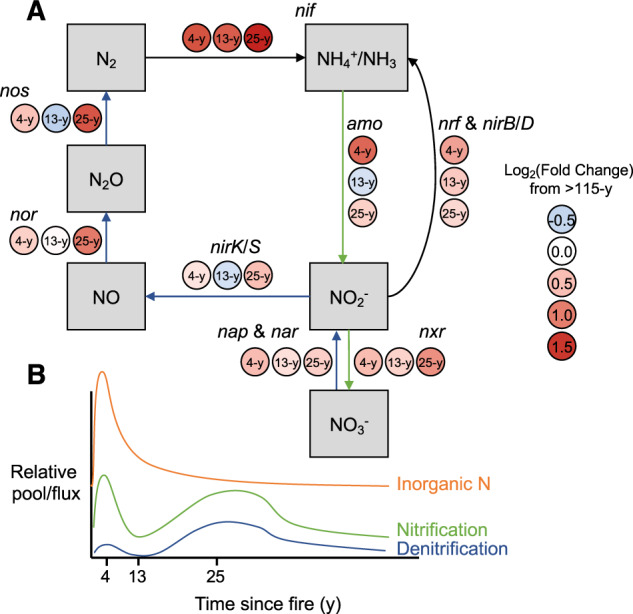
Nitrogen (N) cycling potential of the Central Sierra Nevada Fire Chronosequence. For each pathway, colored circles represent average log_2_(fold change) among sites relative to the >115 y site (**A**). Key: *amo*—ammonium monooxygenase (EC 1.14.99.39), *nap*—nitrate reductase (EC 1.7.1.2), *nar*—nitrate reductase (EC 1.7.99.4), *nif*—nitrogenase (EC 1.18.6.1), *nirB*/*D*—nitrite reductase large/small subunit (EC 1.7.1.15), *nirK*/*S*, *nor*—nitrite reductase precursor (EC 1.7.2.1), *nos*—nitric oxide synthase (EC 1.14.13.39), *nrf*—cytochrome c nitrite reductase (EC (1.7.2.2), *nxr*—nitrite oxidoreductase (EC 1.7.5.1). Conceptual model of N cycling as a function of time since fire (**B**). Lines represent relative differences in inorganic N, the relative genetic potential for nitrification, and the relative genetic potential for denitrification with time since fire. The absolute value at each point on these lines is inconsequential, and as such, meaning is derived from the relative pattern with time since fire.

Given that across many terrestrial ecosystems N is a limiting nutrient for plant productivity [54], microbial N cycling likely influences ecosystem recovery. Generally, the relative genetic capacity for N cycling processes (i.e., the relative abundances of key N cycle genes) was higher during the first three decades post-fire compared to the late-successional site (Fig. 5A). This was most evident for the relative genetic capacity for biological N fixation, which was, on average, 2.7 times higher 4 to 25 y after fire compared to the late successional site (Kruskal–Wallis: *p* = 0.023). This genetic pattern was corroborated by plant cover type measurements, showing that symbiotic N-fixing plants (e.g., *Ceonothus* spp., *Chambaethia* spp.) made up one to two thirds of the vegetative cover during this successional period (Fig. 1B). However, aside from N fixation, each site also had a specific genetic N cycling signature corresponding to the edaphic and environmental conditions associated with post-fire ecosystem succession. For example, the relative genetic capacity for ammonia oxidation differed among the sites (*p* = 0.016) and was highest 4 y after fire (Fig. 5A). Additionally, the relative genetic capacity for nitrate and nitrous oxide reduction, key pathways for denitrification, differed across the sites (nitrate reduction: *p* = 0.043, nitrous oxide reduction: *p* = 0.015) and were highest in the 25 y post-fire site (over twice as high as the late-successional site, Fig. 5A). Taken together, these results suggest that within the first decade of succession after high-severity fire, the relative genetic capacity for nitrification is relatively high, and this pool of nitrate is depleted as the relative genetic capacity for denitrification increases with time since fire (Fig. S6). Such findings are broadly consistent with field measurements from multiple ecosystems showing that nitrification is enhanced within the first decades after fire thus providing the genetic underpinning of these biogeochemical observations [13, 15, 55].

Successional changes in the relative genetic capacity of dominant N cycling processes were related to inorganic N availability and time since fire. While the relative genetic capacity of these different N cycling genes differed with time since fire, biomass-normalized genetic capacity for all measured N cycling processes were unrelated to time since fire and inorganic N availability (*p* > 0.05). This result is likely due to differences in activity of these functional genes. Possibly, when certain N cycling genes are relatively enriched due to favorable environmental conditions, their activity also increases. Therefore, we synthesize the relationship between inorganic N availability, other environmental factors such as C and O_2_ availability, and the relative genetic capacity of N cycling processes, rather biomass-normalized genetic capacity in a conceptual model (Fig. 5B).

Within the first decade after disturbance, N cycling is characterized by a relatively high degree of nitrification, possibly due to low plant N uptake, low microbial immobilization of ammonium (NH_4_^+^) tied to low C availability [56], and high N fixation soon after fire due to the increased abundance of N-fixing shrubs (Figs. 1B and 5A). Additionally, the genetic capacity for denitrification is relatively low, and nitrate (NO_3_^−^) accumulates (Fig. S6) immediately after fire, where low C availability and potential increases in O_2_ availability [57] are limiting environmental factors. During the second decade of succession, genes for nitrification are relatively less abundant (Fig. 5A), possibly because of increased plant competition for NH_4_^+^, C availability, and microbial immobilization of NH_4_^+^ [44, 58]. This results in relatively less N for downstream N cycling processes, and the relative genetic capacity for these processes decreases as well (Fig. 5A). However, as time since fire progresses, plant growth and N uptake likely slow due to light and moisture limitations [29]. This reduces the competition for NH_4_^+^, favoring nitrification and subsequently denitrification; as a result, the relative genetic capacity for these processes increases (Fig. 5A). Thus, this balance of relatively high genetic capacity for nitrification and denitrification dictated by these environmental conditions maintains relatively low levels of NO_3_^−^ (Fig. S6). These results demonstrate the relationships between soil microbial communities, environmental factors, and N cycling processes and provide the much-needed biological basis for previously published biogeochemical measurements [13], linking microbial genetics to ecosystem function. Hence, the succession of post-fire microbial communities, in part, determines the nutritional status of the soil and shapes the trajectory of post-disturbance ecosystem recovery.

### Microbes at different successional states are taxonomically conserved and are adapted to post-fire conditions

**Fig. 6:**
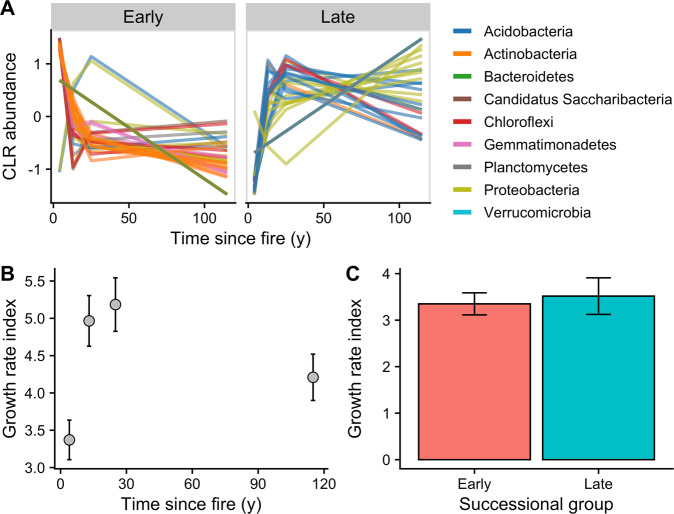
Taxonomy and estimated growth rates of early and late successional metagenome-assembled genomes (MAGs). **A**The MAGs were classified into early or late-successional groups indicated by their center log-ratio (CLR) transformed abundance with time since fire. **B** Mean (±standard error, *n* = 4) growth rate index of MAGs as a function of time since fire. **C** Mean (±standard error) growth rate index across all time points of MAGs classified as either early or late-successional (early: *n* = 82, late: *n* = 59).

We recovered 205 MAGs, representing about 20% of the metagenomic reads, to evaluate genome-resolved differences among successional sites. We found that 82 MAGs decreased in abundance with time since fire, which we operationally classified as early successional and 59 MAGs increased in abundance with time since fire, which we classified as late-successional (Fig. 6A; 64 MAGs did not show any clear trend, Table S5).

The grouping of early and late successional MAGs was taxonomically conserved (Fig. 6A). The pattern was consistent with our 16S rRNA gene and shotgun metagenome phylum profiles, with increasing Proteobacteria and Acidobacteria and decreasing Actinobacteria relative abundances with time since fire (Fig. S1). This was particularly true for the genus *Arthrobacter* (Fig. S7), which is corroborated by numerous previous studies spanning three continents [5, 14, 59, 60]. Taxonomic shifts in the MAG, shotgun metagenome, and amplicon datasets are consistent with previous studies of changes in soil microbial communities after wildfire in various ecosystems [5, 59], suggesting that many patterns of microbial community change post-fire are generalizable at least at the phylum level.

It has been previously shown that microbes 1 to 2 y after disturbance have relatively faster growth rates compared to their late successional counterparts [30]. However, many of these measurements have been based on 16S rRNA gene copy number [5], which correlate with maximum growth rate, but do not necessarily relate to actualized growth rates [61]. To test if growth rates changed over time across our successional gradient, we calculated estimated in situ growth rates for each MAG across the successional sites using peak to trough coverage ratios (PTR) [62]. Estimated growth rates for individual MAGs changed with time since fire (Mixed-effects model with MAG identity as random effect: *p* = 0.002). For instance, estimated growth rates at the 13- and 25-y sites were about 1.5 times greater than those at the 4-y site (Fig. 6B). However, we did not find differences in average estimated growth rates between our early and late successional MAGs (Wilcox rank sum test: *p* = 1.000, Fig. 6C). This could be due to the Gaussian nature of growth rates with time since fire, where growth rates are relatively lower at both 4 y and >115 y after fire compared to periods 1 to 3 decades after fire (Fig. 6B). Higher growth rates in the sites 1 to 3 decades after fire are likely the result of relatively higher soil C (compared to the 4-y site, Table S4) and nutrient (compared to the >115-y site, Fig. S6) availability that supports faster growth. The traditional paradigm is that early in succession (1 to 2 years after disturbance), microbes are fast-growing, ruderal species [30]. However, our results suggest that mid-successional microbial communities (1–3 decades after disturbance) also have relatively fast growth rates once microbial substrates have increased. These results do not necessarily discredit the previous paradigm that early successional microbes have fast growth rates [30], as 4 y after fire (our earliest post-fire measurements) may have been too late to capture many of the earliest post-fire colonizers. Hence, further studies of microbial growth rates using PTR and physiological methods (e.g., quantitative stable isotope probing) within the first year after fire are necessary in understanding early post-fire microbial succession.

**Fig. 7:**
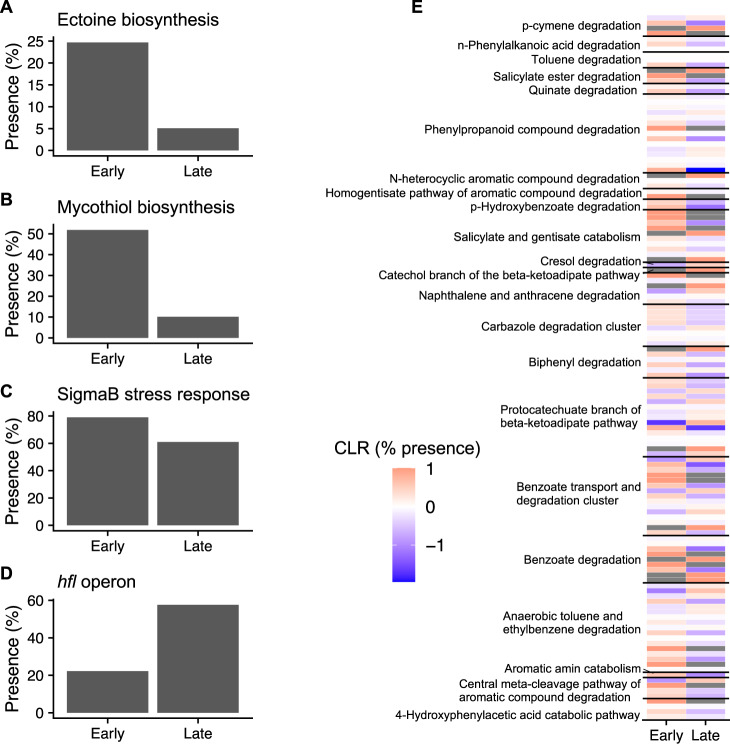
Traits of early and late successional metagenome-assembled genomes (MAGs). **A–D** Percent presence of stress-related genes. **E** Heatmap of differentially present genes (*χ*^2^ test—*p* < 0.05, early: *n* = 82, late: *n* = 59) within major pathways of aromatic carbon degradation. Colors represent center log ratio (CLR) of percent presence for each gene; gray tiles represent genes that were absent in a specific successional group (i.e., CLR = undefined). Genes within major pathways of aromatic carbon degradation are described in Table S5.

Early successional MAGs were enriched in stress response genes compared to late-successional MAGs (Fig. 7A–C), which could allow them to survive in stressful post-fire environments. For example, early successional MAGs were about five times as likely to have genes encoding for ectoine and mycothiol biosynthesis (Chi-square test: *p* < 0.05, Fig. 7A, B). Ectoine is released as a stress protectant, and it provides osmotic stress tolerance, confers protection against rapid temperature fluctuations, and protects DNA from ionizing radiation damage [63], all of which occur in post-fire environments with tree canopy loss [29, 64]. Mycothiol is a glutathione analog found in Actinobacteria and acts as a thiol buffer, which maintains a reducing environment within the cell [65]. The early successional sites may have been highly oxidative due to lower water holding capacities (Table S4) and enriched metal oxides found in deposited ash [36]. Therefore, mycothiol production may have been necessary to deal with this redox stress, especially as 38 of 59 early successional MAGs belonged to the phylum Actinobacteria, which do not synthesize glutathione [65]. Furthermore, genes involved in the SigmaB stress response were also significantly more present in early successional MAGs compared to late-successional MAGs (*p* = 0.033, Fig. 7C). SigmaB is a general, non-specific stress regulon that activates a variety of genes in response to diverse stresses, including salt, heat, and osmotic stresses [66, 67]. Alternatively, late-successional MAGs were enriched in the *hfl* operon (*p* < 0.001, Fig. 7D), which includes *hflX*, a gene that confers resistance to macrolide antibiotics in *Mycobacterium abscessus* [68]. Cell defense against antimicrobials may be selected for as competition among microbial populations increases with time since fire. Taken together, enrichment of these stress response genes in early successional MAGs suggests that adaptations leading to improving cell fitness are key to surviving early in succession in highly oxidative and desiccated post-fire soils.

Early successional MAGs were also enriched in genes encoding for aromatic C degradation, which allows them to exploit common post-fire C substrates and degrade potential xenobiotic compounds in the soil [69]. Polyaromatic C compounds are produced via pyrolysis and incomplete combustion of organic matter and are found in high abundances in post-fire environments [70]. Notably, these MAGs were commonly capable of aromatic amine catabolism, p-hydroxybenzoate degradation, and n-phenylalkanoic acid degradation (Fig. 7E, Table S5). This is similar to recent findings that *Pyronema*, a common pyrophilic fungus, is capable of degrading pyC through catechol and quinate degradation [71], functions that were also prevalent in many of our early successional MAGs (Fig. 7E, Table S5). Hence, our results show that early successional microorganisms are especially adapted to degrade pyC, potentially increasing their fitness over microorganisms without this capacity. As 54–109 Pg of pyC is stored in soils [70], the ability of the microbial community to degrade pyC compounds may play a critical role in determining the stability of the post-fire soil C pool.

Because our results suggest that post-fire microbes are well-adapted to their environment, post-fire microbial inoculation strategies to enhance management objectives [72] should prioritize microbes that are capable of tolerating the stressful, post-fire environment. While earlier microbial interventions have had mixed successes [73, 74], our findings that post-fire microbes are highly adapted to stressful and oxidative conditions suggest that many late-successional microbes may not be able to survive in early successional post-fire environments. Therefore, we propose that future research of microbial interventions should focus on promoting native microbial populations cultured or extracted from early successional post-fire soils with robust stress response systems and the capacity to synthesize stress protectants, such as those highlighted in this study.

## Conclusion

Our results show that the recovery of the ecosystem from high-severity fire can take several decades and is associated with the succession of the microbial community. Specifically, we find that changes in microbial genetic potential reflects bioavailability of soil N, which likely influences the establishment and regeneration of plant communities. We also show that after 4 y since fire, microbial recovery is not dominated by fast-growing, ruderal species; rather, after 4 y, microbial assembly is governed mainly by deterministic selection in the stressful, C-limited post-fire soil environment typical of the early and middle stages of ecosystem succession. As such, future studies should investigate how management of post-fire soils can create conditions leading to the recovery of beneficial microbes (e.g., N-fixing bacteria, mycorrhizal fungi), such as the rapid re-establishment of the native plant community. In certain circumstances, broadcast mulch additions [75] may be useful to alleviate C limitation and drought stress on the microbial community, leading to increased microbial resilience and N stability [44]. However, assisted colonization of microbial inoculants may be, in most cases, unnecessary, as dispersal limitation played only a minor role in prokaryote recovery. Mycorrhizal inoculations may still be useful; however, target fungal symbionts should have robust stress response systems. As wildfire size, severity, and frequency are increasing worldwide [2], increasing resilience of forest ecosystems to fire and developing strategies to aid recovery will become critical in post-fire restoration and the maintenance of valuable forest ecosystem services.

## Methods

### Experimental design

This study was conducted on the Eldorado National Forest, located in the Central Sierra Nevada of California (Fig. 1A). We sampled in areas of varying time since stand-replacing wildfire using a fire chronosequence established within the South Fork of the American River Watershed. For a full description of the chronosequence, see Bohlman et al. [16], Dove et al. [13], and Dove [76]. Briefly, the fire sites are as follows: King Fire (4-y since fire), Freds Fire (13-y since fire), and Cleveland Fire (25-y since fire). We incorporated sites throughout the study area that had not burned since at least 1908 [77], which is the maximum period for which we know that no recorded burning occurred in this region. We operationally defined this as our late successional site (>115-y since fire). We controlled for pre-fire vegetation, elevation, slope, aspect, burn severity, post-fire management, and USDA Soil Taxonomy (suborder) for all plots [13]. Each site consisted of six to eight plots separated by at least 150 m, and we sampled the top 5 cm (excluding the organic horizon) under each cover type present. For further detail, see the Supplementary methods and Table S4.

### DNA extraction and bioinformatics

We extracted total soil DNA (0.25 g of field moist soil) using the MoBio PowerSoil DNA isolation kit (Carlsbad, CA). Amplicon libraries were prepared and sequenced at the Department of Energy Joint Genome Institute (Berkeley, CA, USA), targeting the V4 region of the 16S rRNA gene [78] and the ITS2 region [79]. Sequences were grouped into OTUs based on 97% sequence identity, and chimeric sequences were removed using the USEARCH and UPARSE algorithms [80]. Taxonomy was annotated to the SILVA 132 database [81] for 16S rRNA genes, and to the UNITE database for ITS2 region [82] using USEARCH.

We chose four plots randomly from each fire site for shotgun metagenomic sequencing because cost constraints prevented us from sequencing all samples (16 total metagenomes). These metagenome samples were prepared by compositing DNA (on a mass basis) from all samples under different cover types within a plot in proportions relative to the percentage of that cover type within the plot. Metagenome libraries were sequenced on the NovaSeq sequencer (Illumina, San Diego, CA, USA) at the Joint Genome Institute (Berkeley, CA, USA). For further detail on library preparation, sequencing, and bioinformatic analysis, see the Supplementary methods.

### Statistical analyses

Differences in microbial compositions were determined by PerMANOVA [83] using Bray-Curtis distances [84] on proportionally normalized data. We conducted distance-based redundancy analysis using edaphic data from Dove et al. [13] to assess the variation of the microbial community explained solely by soil physical and chemical properties. Assembly processes were assessed using a null modeling approach following Stegen et al. [18] for the whole-community analysis and following Ning et al. [37] for the phylogenetic bin analysis. Both approaches used the entire dataset to define the null model parameters. Differences in CAZy abundance (including different functional types), N cycling genes, and MAG growth rates with time since fire were determined by Spearman correlations, Kruskal–Wallis tests, and Wilcoxon Rank sum tests on data normalized by Prodigal predicted amino acid coding reads [85]. To assess differences in the composition of CAZy genes with time since fire, we followed the same approach as for amplicons except, instead of proportionally normalizing data, data were normalized by predicted amino acid coding reads. We classified MAGs as early or late-successional based on splines of their center log-ratio abundance with time since fire, using sparse principal components analysis [86], and differences in gene presence between early and late successional MAGs were determined by the Chi-square test of independence. For further details on statistical analysis, see the Supplementary methods.

## Supplementary information

Supplemental Material

Supplemental Tables 5 and 6

## Data Availability

Amplicon sequence data are deposited in the JGI Genome Portal under project ID 1188685. Shotgun metagenome sequence data are deposited at the Sequence Read Archive under the following Project IDs: 566978, 566979, 566980, 566981, 566982, 566983, 621559, 621560, 621561, 621562, 621563, 621564, 621565, 621566, 621567, and 621568. Metagenome-assembled-genomes are available through KBase narrative (KBase account required): https://narrative.kbase.us/narrative/92072.
