# Supplementary material for: Ecological and genomic responses of soil microbiomes to high-severity wildfire: linking community assembly to functional potential
Source: ISME J. 2022 Apr 16;16(7):1853–63. doi: 10.1038/s41396-022-01232-9 (PMC9213548; doi:10.1038/s41396-022-01232-9)
Supplement: Supplementary file 1 — Supplemental Material [file 41396_2022_1232_MOESM1_ESM.pdf]

Supplementary Information for “Ecological and genomic response of soil microbiomes to wildfire: linking fundamental community assembly processes to ecosystem function”

Nicholas C. Dove [1,2*], Neslihan Taş [3], and Stephen C. Hart [4]

[1] Environmental Systems Graduate Group, University of California, Merced, CA USA
[2] Biosciences Division, Oak Ridge National Laboratory, Oak Ridge, TN USA
[3] Climate & Ecosystems Division, Lawrence Berkeley National Laboratory, Berkeley, CA USA
[4] Department of Life & Environmental Sciences and Sierra Nevada Research Institute, University of California, Merced, CA USA

*Corresponding author: Nicholas C. Dove
Email: ndove7@gmail.com

SUPPLEMENTARY METHODS

Site description, experimental design, and soil sampling

This study was conducted on the Eldorado National Forest, which is located in the Central Sierra Nevada of California, an area historically fire-suppressed like much of western North America (Figure 1A) [1]. We sampled in areas of varying time since stand-replacing wildfire using a fire chronosequence established within the South Fork of the American River Watershed. For a full description of the chronosequence, see Bohlman et al. [2], Dove et al. [3], and Dove [4]. Briefly, the fire sites are as follows: King Fire (4-y post fire), Freds Fire (13-y post fire), and Cleveland Fire (25-y post fire). We incorporated sites throughout the study area that had not burned since at least 1908 [1], which is the maximum period for which we know that no recorded burning occurred in this region. We operationally defined this as our late-successional site (> 115-y post fire). We controlled for pre-fire vegetation, elevation, slope, aspect, burn severity, post-fire management, and USDA Soil Taxonomy (suborder) for all plots [3]. All soils are in the suborder Xerepts, with either an umbric or ochric epipedon.

Each site consisted of six to eight plots separated by at least 150 m (the 13-y site had only six plots due to sampling area constraints; all other sites had eight plots). Plot-centers were chosen randomly *a priori* using a GIS layer of appropriate site polygons (i.e., similar soils, elevation, aspect, burn severity, and management). Each plot was defined by a 5-m radius from plot-center. Within each plot, we sampled one point under each of the available lowest-stratum cover types. Cover types were tree (e.g., *Abies concolor*, *Pinus ponderosa*, *Quercus* spp.), seedling (e.g., *Pinus ponderosa*), shrub (*Arctostaphylos* spp., *Salix* spp.), nitrogen-fixing plant (*Ceonothus* spp., *Chamaebatia*

foliolosa), herbaceous (e.g., *Carex* spp., Poaceae), and bare soil. When multiple representatives of the same cover type occurred within a plot (which occurred frequently), we sampled under the representative closest to plot-center.

We sampled mineral soil June 8-15, 2017. The organic horizon was removed with a sterile, gloved hand, and a 2-cm diameter soil corer (sterilized with 10% bleach followed by 70% ethanol) was used to sample the top 5 cm of mineral soil, where we expected the greatest impacts of fire, at each point. We did not sample the organic horizon because many of the 4- and 13-y plots did not have this horizon. To collect enough soil from each point (~100 g), we took and composited multiple (~10) cores within a 20-cm diameter area, within a given cover type stratum. Soil samples were placed in a sterile bag, immediately placed on Blue Ice® (4 °C) and transferred to dry ice (-80 °C) within 3 h [4].

DNA extraction

We extracted total soil DNA (0.25 g of field moist soil) using the MoBio PowerSoil DNA isolation kit (Carlsbad, CA), following the manufacturer's instructions. We quantified DNA yields using the Quant-it PicoGreen dsDNA assay kit (Invitrogen, Carlsbad, CA).

Amplicon sequencing and analysis

Sample libraries were prepared and sequenced at the Department of Energy Joint Genome Institute (Berkeley, CA, USA). For prokaryotes, 16S rRNA genes were amplified in polymerase chain reactions (PCRs) using primers (515F/806R) that target

the V4 region of the 16S rRNA gene [5]. For fungi, ITS2 regions were amplified in PCR reactions using ITS9f/ITS4R primers [6]. The PCR reactions contained 10 µl 5 PRIME HotMasterMix (Quantabio, Beverly, MA), 1 µl Roche BSA (10 mg/ml), 0.5 µl each of the forward and reverse primers (10 µM final concentration), 1.0 µl genomic DNA (10 ng/reaction), and nuclease-free water in total volume of 25 µl. Reactions were held at 94 °C for 3 min to denature the DNA, followed by amplification for 30 cycles at 94 °C for 45 s, 50 °C for 60 s, and 72 °C for 90 s; a final extension of 10 min at 72 °C was added to ensure complete amplification.

Each sample was amplified in triplicate, combined, and purified using the Agencourt AMPure XP PCR purification system (Beckman Coulter, Brea, CA). Amplicons were pooled (10 ng/sample) and sequenced on one lane of the Illumina Miseq platform (Illumina Inc., San Diego, CA), resulting in 300 bp paired-end reads. Adapters were trimmed and known contaminants were removed using BBDuk kmer filtering (https://jgi.doe.gov/data-and-tools/bbtools/bb-tools-user-guide/bbduk-guide/). Sequences were grouped into operational taxonomic units (OTUs) based on 97% sequence identity, and chimeric sequences were removed using USEARCH [7]. Taxonomy was annotated to the SILVA 132 SSU V4-V5 database [8] for 16S, and to the Unite database for ITS (v7) [9] using USEARCH. Amplicon sequence data are deposited in the JGI Genome Portal under project ID 1188685.

Metagenomic sequencing and analysis

We chose four plots randomly from each fire site for shotgun metagenomic sequencing because cost constraints prevented us from sequencing all samples (16

total metagenomes). These metagenome samples were prepared by compositing DNA (on a mass basis) from all samples under different cover types within a plot in proportions relative to the percentage of that cover type within the plot. Plate-based DNA library preparation for Illumina sequencing was performed on the PerkinElmer Sciclone NGS robotic liquid handling system (Waltham, MA, USA), using Kapa Biosystems library preparation kit (Wilmington, DE, USA). Next, 200 ng of sample DNA was sheared to 300 bp using a Covaris LE220 focused-ultrasonicator (Woburn, MA, USA). The sheared DNA fragments were size-selected by the double Solid Phase Reversible Immobilization (SPRI) method (Beckman Coulter, CA, USA), and then the selected fragments were end-repaired, A-tailed, and ligated with Illumina compatible sequencing adaptors from IDT (Coralville, IA, USA) containing a unique molecular index barcode for each sample library. The prepared library was quantified using KAPA Biosystem's next-generation sequencing library qPCR kit and run on a Roche LightCycler 480 real-time PCR instrument (Basel, Swtizerland). The library was then multiplexed and sequenced on the Illumina NovaSeq sequencer (San Diego, CA, USA) using NovaSeq XP V1 reagent kits and S4 flow cell, following a 2X151 indexed run recipe at the Joint Genome Institute (Berkeley, CA, USA). Shotgun metagenome sequence data are deposited at the Sequence Read Archive under the following Project IDs: 566978, 566979, 566980, 566981, 566982, 566983, 621559, 621560, 621561, 621562, 621563, 621564, 621565, 621566, 621567, and 621568.

Coverage of each metagenome was assessed using Nonpareil (v. 3.3.3) [10]. Sequencing depth averaged 20.2 Gbp and ranged from 12.4 to 28.7 Gbp, and coverage averaged 48.2% and ranged from 36.2% to 70.0%.

Raw reads were trimmed and quality filtered using Trimmomatic (v 0.36, [11], and read taxonomy was classified using Kaiju (v 1.6) [12]. Prodigal (v 2.6) [13] was used to predict coding regions from the reads. The translated proteins from all detected coding regions of each metagenome were annotated by searching against carbohydrate active enzymes using the CAZy database [14, 15] via DIAMOND BLASTp (options: -k 1 -e 1E-5–sensitive) [16]. Nitrogen (N) cycling, methane production and oxidation, and sulfate reduction genes were annotated via hmmer (v 3.1b2) [17] to the Kyoto Encyclopedia of Genes and Genomes (KEGG) database [18]. Gene abundances (gene counts per KEGG Orthology - KO) were normalized to the number of amino acids detected in each metagenome.

Samples were co-assembled using MEGAHIT [19] with a minimum contig length of 1000 bp. Then, each individual sample was mapped back to the MEGAHIT contigs with bbmap [20], and we extracted unmapped reads. Next, unmapped reads were concatenated and re-assembled using SPAdes in the "meta" setting [21]. The newly assembled contig folds were merged with the MEGAHIT contigs. Genome fragments that were larger than 1 kb were clustered into MAGs using MaxBin (v 2.2.5) [22] and MetaBAT2 (v 2.12.1) [23], and MAGs were dereplicated using DASTool (v 1.1.10) [24]. Potential mis-binning was identified with CheckM [25], and bins were further refined to remove contamination following Xue et al. [26]. Genome bin completeness and contamination are reported (Table S6). Each bin was annotated with Kaiju (v 1.6.2) [12] using default parameters, the NCBI nr database, and GTDB-Tk (v 0.3. 2) [27] to classify each contig into a taxonomic rank, from phylum to species. Protein-encoding genes from MAGs were predicted with Prodigal [13], and the resulting nucleotide sequences

were searched against the KEGG database [18] reference sequences using DIAMOND BLASTX [16].

We extracted 49 high-quality (>90% completeness, <10% contamination) and 156 medium-quality (>50% completeness, <11% contamination) draft MAGs from our metagenomes, representing 12 bacterial phyla (Table S6) [28]. The genome bin sizes were between 0.9 and 9.6 Mbp, with a variable GC content ranging from 33-75%. On average, 20.4% of our metagenomic reads could be mapped back to our metagenomes, with MAGs constituting a larger proportion of the reads in the less-diverse, 4-y site (4 y: 26.7%, 13 y: 20.6%, 25 y: 15.2%, >115 y: 18.9%). Metagenome-assembled-genomes are available through KBase narrative (KBase account required): https://narrative.kbase.us/narrative/92072.

Statistical Analyses

All statistical analyses were conducted in R [29] using the lsmeans [30], mixOmics [31], nlme [32], phyloseq [33], and vegan [34] packages. Significance was determined *a priori* at the $\alpha = 0.05$ level for all statistical tests.

Differences in the prokaryotic (16S rRNA gene) and fungal (ITS region) compositions were determined by PerMANOVA [35] using Bray-Curtis distances [36] on proportionally normalized data. We conducted distance-based redundancy analysis (dbRDA) using edaphic data from Dove et al. [3] to assess the variation of the microbial community explained solely by soil physical and chemical properties. Differences in the richness and abundance of ectomycorrhizal and arbuscular mycorrhizal fungi

(annotated using FUNGuild) [37] among cover types and with time since fire were determined using ANOVA and linear regression.

Assembly processes were assessed using null-modeling approaches following Stegen et al. [38] and Ning et al. [39]. Assembly processes are broadly categorized as: 1) variable selection, whereby selective processes lead to disparate microbial communities; 2) homogenous selection, whereby selective processes lead to similar microbial communities; 3) dispersal limitation, whereby limitations to dispersal allow ecological drift to lead to disparate microbial communities; and 4) homogenizing dispersal, whereby high rates of dispersal lead to similar microbial communities.

The Stegen et al. [38] approach uses both phylogenetic and taxonomic turnover to classify the dominant assembly process in pairwise sample comparisons. We used the between-community version of the (abundance-weighted) β-mean-nearest taxon distance (βMNTD) to characterize phylogenetic turnover [40]. Observed βMNTDs were then compared to a null model distribution of βMNTDs generated from 999 null model expectations (i.e., randomized taxa reshuffling among phylogenetic tree tips). The difference between observed βMNTD and the mean of the null distribution was measured in units of standard deviation (of the null distribution), and it is referred to as the β-nearest taxon index (βNTI). Values of $\beta NTI \geq 2$ signify variable selection as the dominant assembly process, and βNTI values ≤ -2 signify homogenous selection as the dominant assembly process [38]. Taxonomic turnover was used to define the dominant assembly processes of $|\beta NTI$ values$| < 2$. For taxonomic turnover, we use the Raup Crick index [41] modified to include species abundances [38]. We calculated null models using probability-based randomization. Null model microbial compositions were

assembled for each sample by randomly sampling from the total OTU pool in proportion to their occupancy and abundance across all samples, maintaining similar levels of alpha diversity. The community composition for each sample was probabilistically generated 999 times. For each iteration of the null model, a pairwise Bray-Curtis dissimilarity matrix was calculated. For each pair of samples, the proportion of null iterations in which the index was smaller than or equal to the observed Bray-Curtis dissimilarity index was our resulting metric. We standardized this metric (RC_{Bray}) to range from -1 to 1 by subtracting 0.5 and multiplying by 2 [41]. Values of $|\beta NTI| < 2$ and $RC_{Bray} \geq 0.95$ signify dispersal limitation, and values of $|\beta NTI| < 2$ and $RC_{Bray} \leq -0.95$ signify homogenizing dispersal [38]. Values of $|\beta NTI| < 2$ and $|RC_{Bray}| < 0.95$ signify weak selection and moderate levels of dispersal such that no process dominates assembly and are therefore classified as “undominated” [42].

The Ning et al. [39] approach uses the iCAMP R package and is similar to the Stegen et al. [38] approach, except assembly processes are calculated for phylogenetic bins of closely related taxa. For this, default parameters were used. We used the taxa.binphy.big() function to determine optimal minimal phylogenetic difference for a significant phylogenetic signal (0.2) and phylogenetic bin size limit (24) with the environmental parameters soil organic carbon, N, and pH from Dove et al. [3].

Differences in CAZy abundance (including different functional types) and N cycling genes with time since fire were determined by linear regression and ANOVA on data normalized by Prodigal predicted amino acid coding reads [13]. To assess differences in the composition of CAZy genes with time since fire, we followed the same approach as for amplicons except, instead of proportionally normalizing data, data were

normalized by predicted amino acid coding reads. We classified MAGs as early- or late-successional based on splines of their center log-ratio abundance with time since fire, using sparse principal components analysis [31].

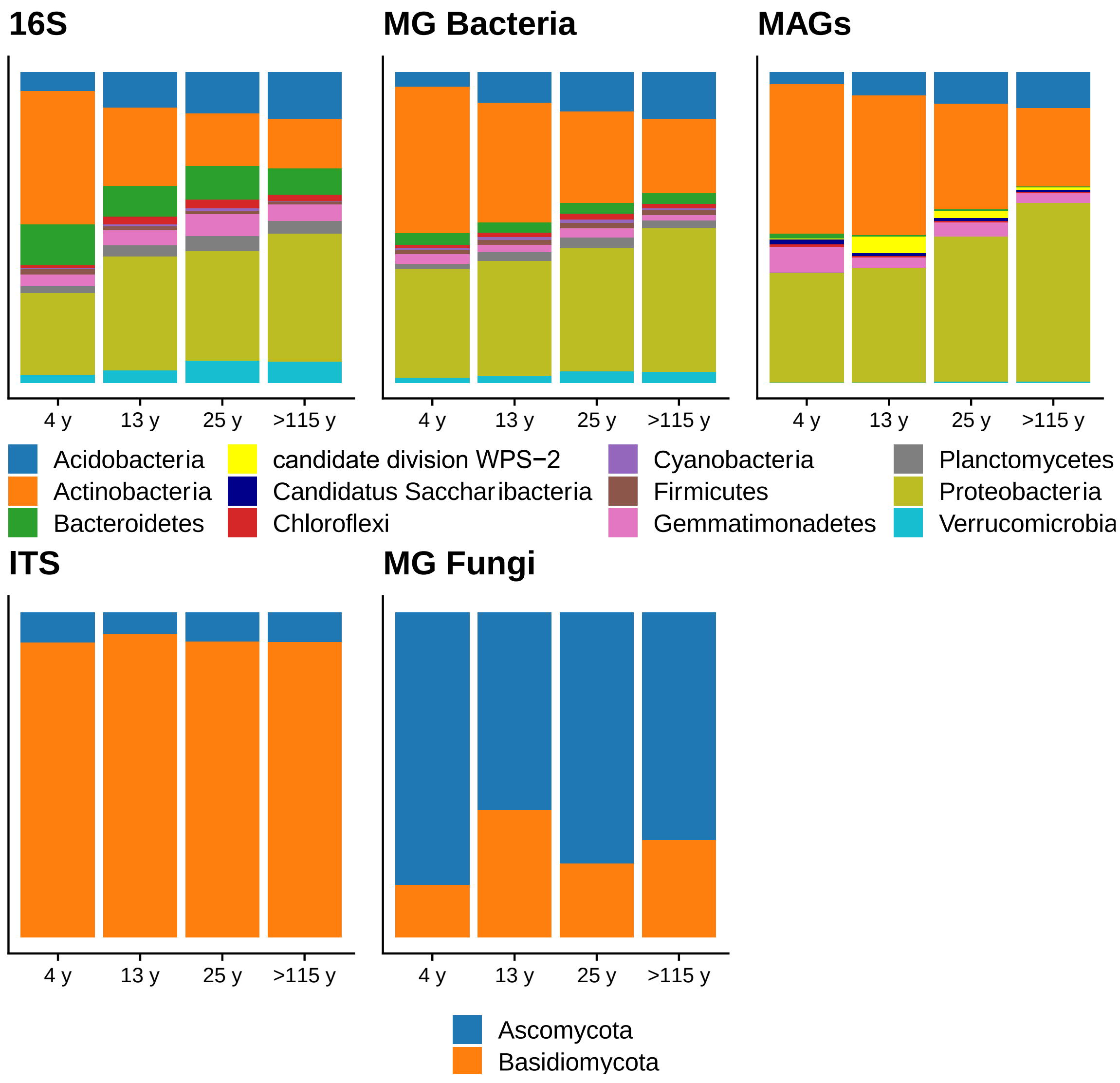

Fig. S1. The relative abundance of the most abundant bacterial and two most abundant fungal phyla across the four fire sites. At the phylum-level, 16S, bacterial metagenomic (MG Bacteria), and metagenome-assembled genome (MAG) read profiles follow similar patterns, but ITS and fungal metagenomic (MG Fungi) read profiles were uncorrelated.

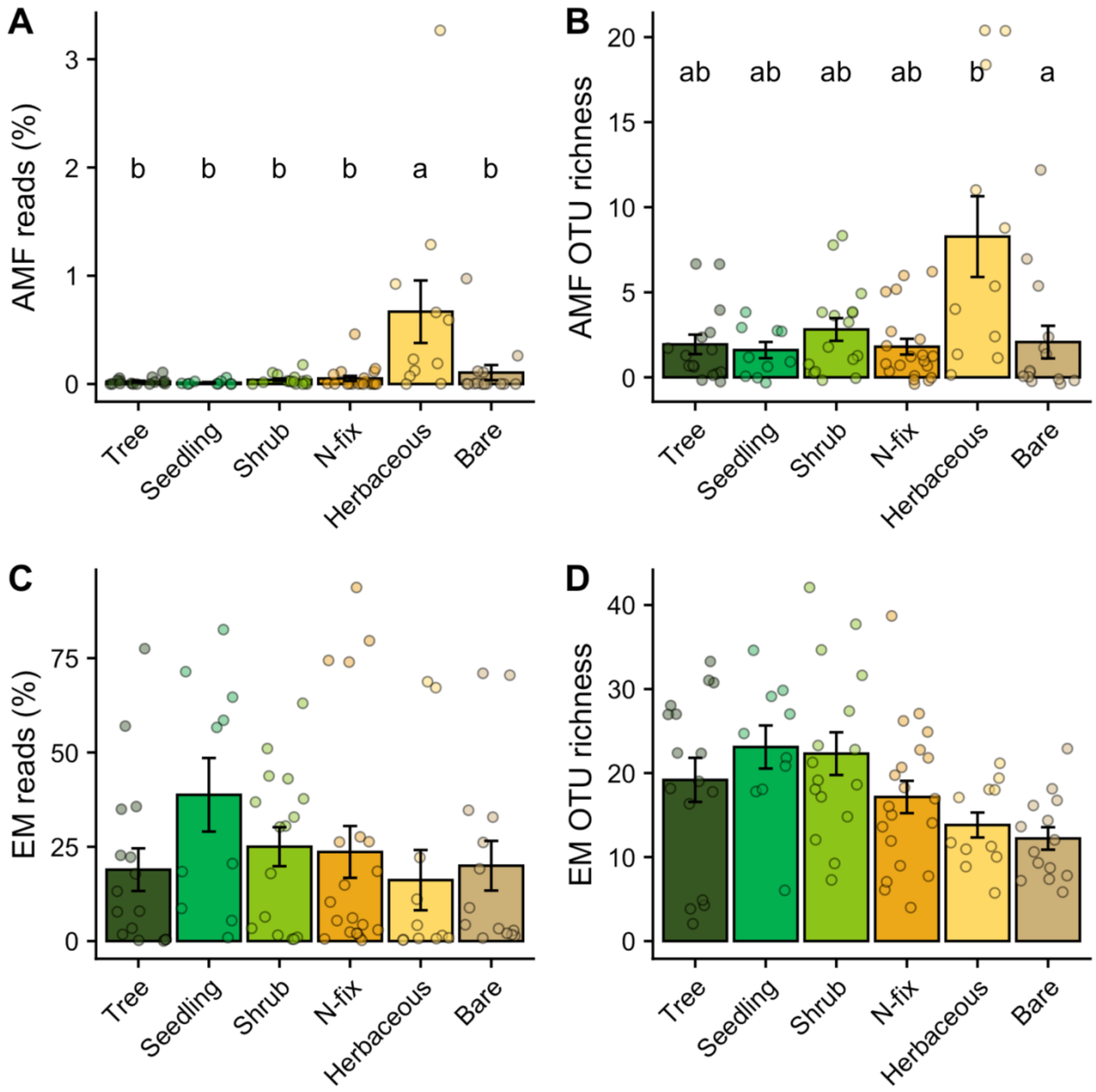

Fig. S2. Mean (+/- standard error of the mean) arbuscular mycorrhizal fungi (AMF) relative abundance (**A**), AMF operational taxonomic unit (OTU) richness (**B**), ectomycorrhizal (EM) relative abundance (**C**), and EM OTU richness (**D**) among vegetative cover types. Points represent samples across all sites. Letters above bars represent significant differences ($p < 0.05$) using Tukey's Test of Honest Significant Differences.

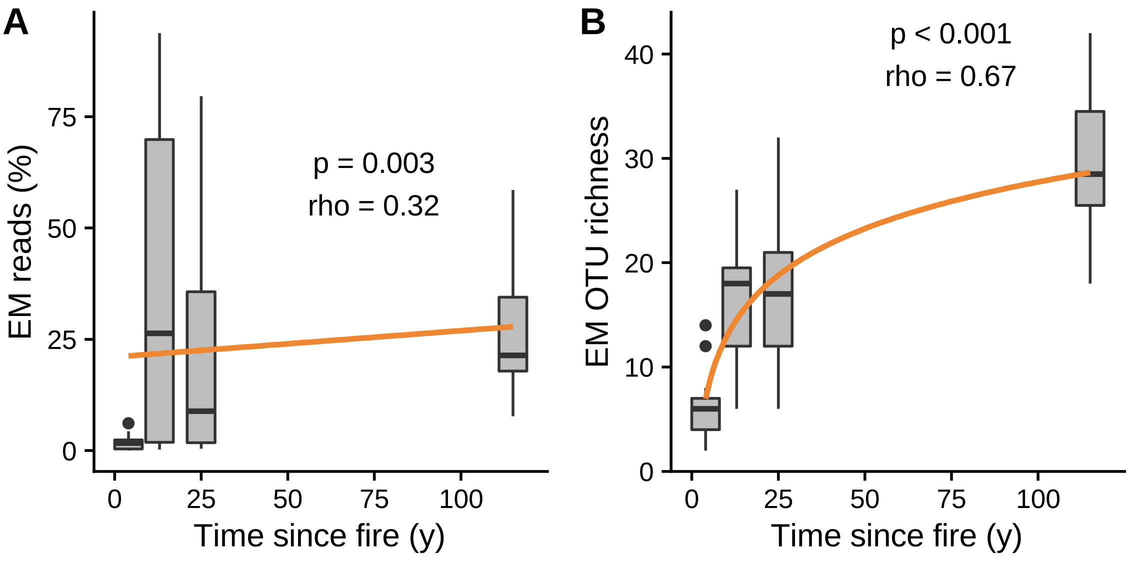

Fig.S3. Boxplots representing ectomycorrhizal (EM) relative abundance (**A**) and operational taxonomic unit (OTU) richness (**B**) plotted as a function of time since fire. The orange line represents the best-fit regression.

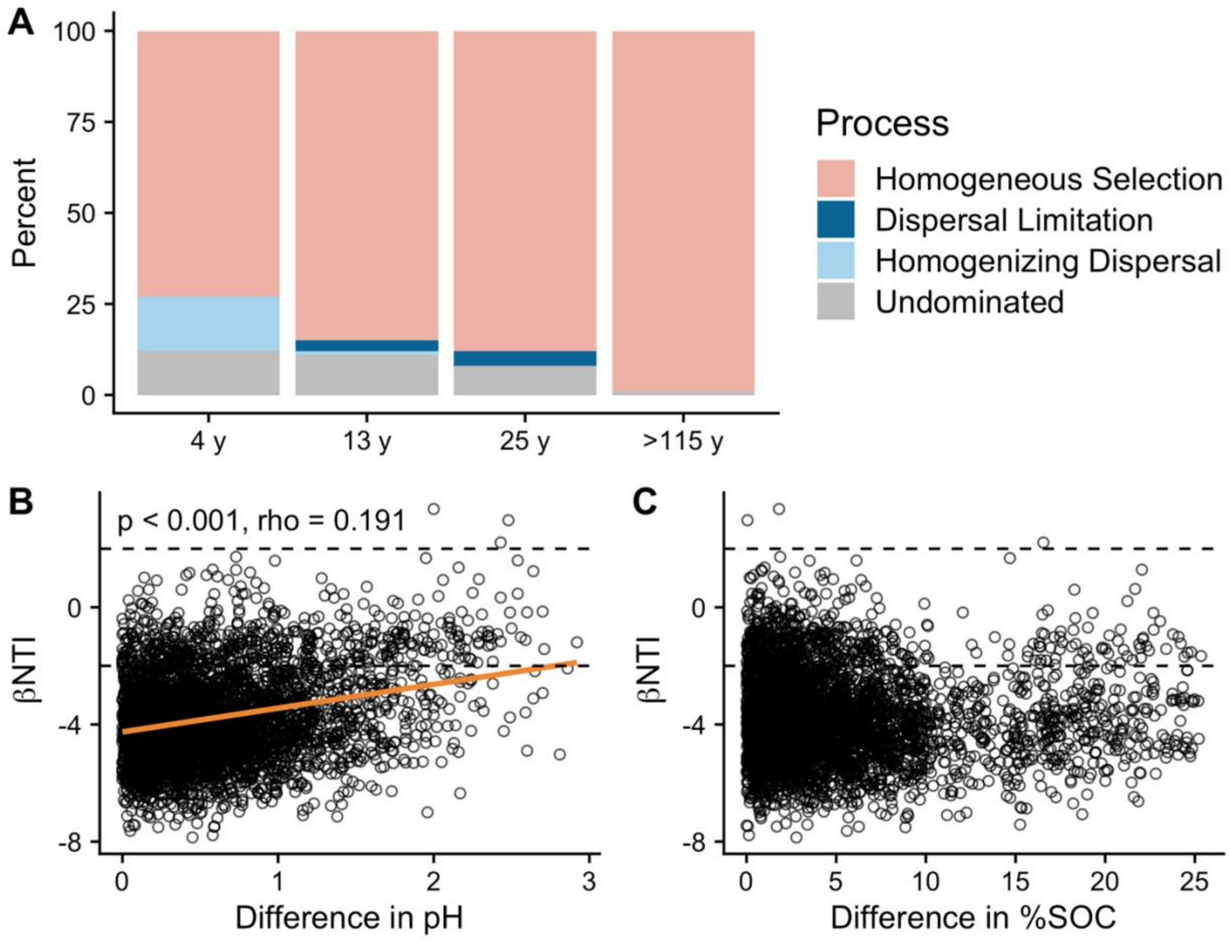

Fig. S4. Relative dominance of assembly processes for prokaryotes within each time point (variable selection was not detected, **A**). Correlations between βNTI and pairwise differences in pH (**B**) and percent soil organic carbon (SOC, **C**). The orange line represents the best-fit regression. Dashed horizontal lines at βNTI = -2 (homogeneous selection) and βNTI = 2 (variable selection) represent thresholds for assembly processes.

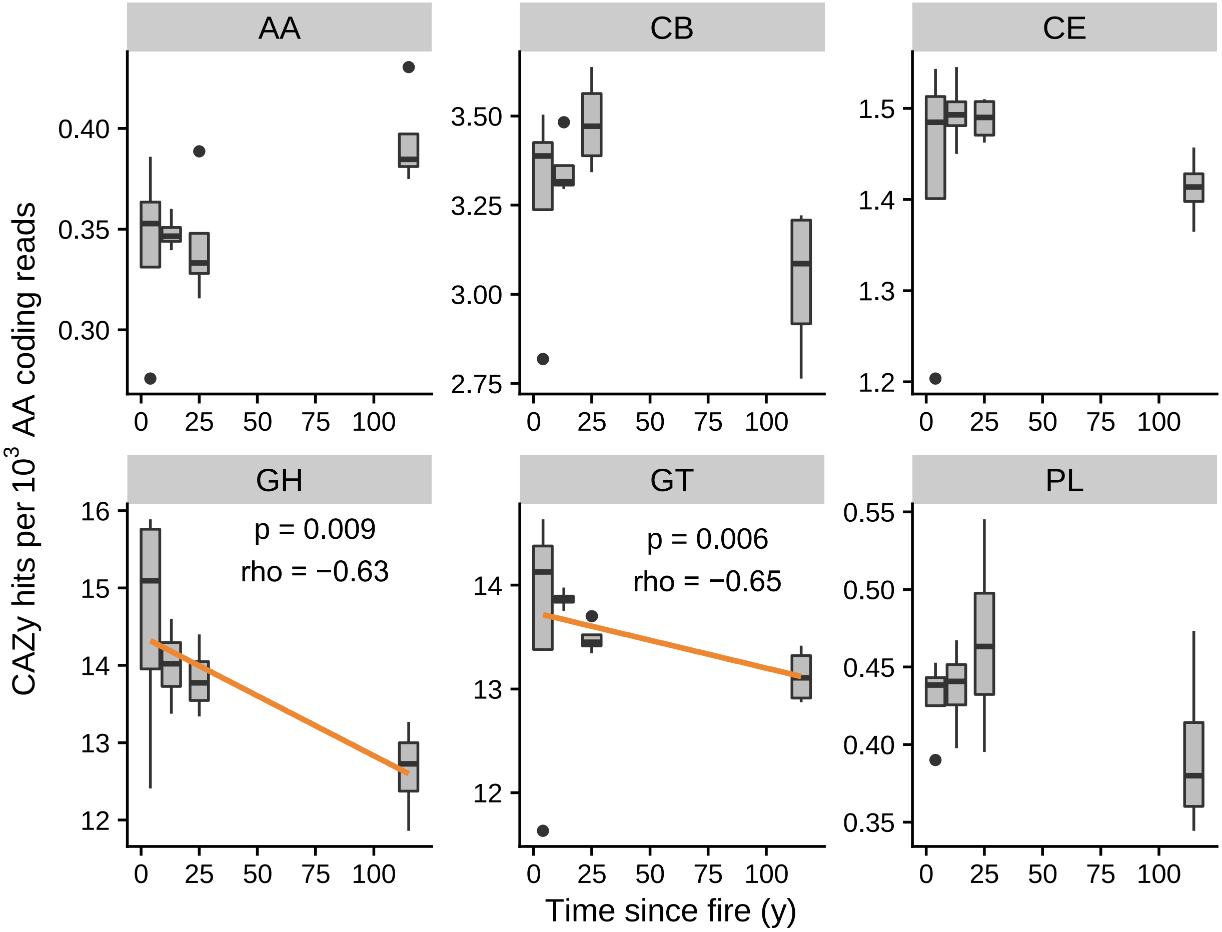

Fig. S5. Boxplots representing abundance of carbohydrate-active enzyme (CAZy; [14, 15]) families plotted as a function of time since fire. Abundances were normalized by amino acid (AA) coding reads. The orange lines represent the best-fit linear regressions where significant (Spearman correlation: $p < 0.05$) relationships occur. Note different Y-axis scales. Key: AA = Auxiliery Activities, CB = Carbohydrate Binding Modules, CE = Carbohydrate Esterases, GH = Glycoside Hydrolases, GT = GlycosylTransferases, PL = Pectin Lyases.

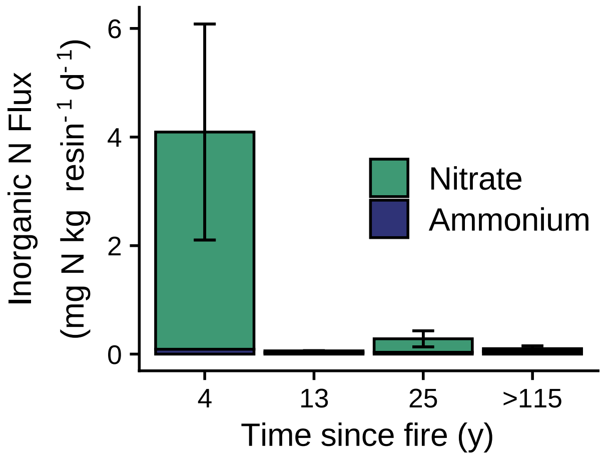

Fig. S6. Mean (+/- standard error) inorganic nitrogen (N) flux across the four chronosequence sites. Fluxes were measured using ion exchange resin bags incubated *in situ* from November 2016 through June 2017 (n = 8). Data are reproduced from Dove et al. [3].

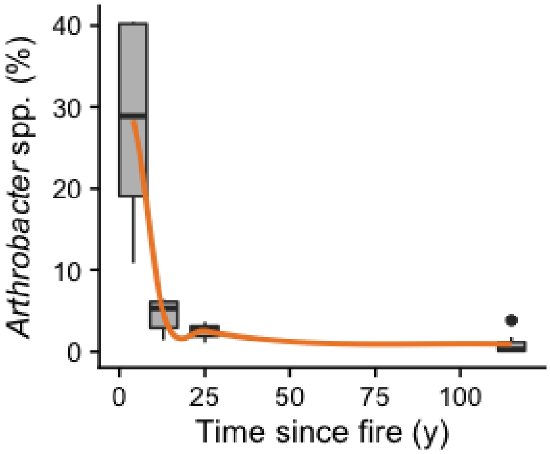

Fig. S7. *Arthrobacter* spp. relative abundance decreases with time since fire. Boxplots represent relative abundance of *Arthrobacter* spp. in samples from different sites, and the orange line represents the best-fit local weighted regression.

SUPPLEMENTARY TABLES

Table S1. Summary statistics of PerMANOVAs for multiple comparisons among fire sites.

Archaea/Bacteria (16S)

Contrast		*F*-value	*p*-value*	R^2
4 y	13 y	15.91	< 0.001	0.319
	25 y	23.97	< 0.001	0.353
	>115 y	26.96	< 0.001	0.473
13 y	25 y	5.71	< 0.001	0.096
	>115 y	12.61	< 0.001	0.240
25 y	>115 y	10.74	< 0.001	0.177

Fungi (ITS)

Contrast		*F*-value	*p*-value*	R^2
4 y	13 y	3.84	< 0.001	0.102
	25 y	5.51	< 0.001	0.111
	>115 y	9.79	< 0.001	0.252
13 y	25 y	3.47	< 0.001	0.060
	>115 y	6.14	< 0.001	0.136
25 y	>115 y	8.47	< 0.001	0.147

*The *p*-values are corrected by the false discovery rate [43].

Table S2. Summary statistics of PerMANOVAs for multiple comparisons among vegetative cover types (bolded values indicate significant differences (p < 0.05)).

Contrast		Archaea/Bacteria (16S)			Fungi (ITS)		
		F-value	*p*-value*	R^2	*F*-value	*p*-value*	R^2
Tree	Seedling	1.10	1.000	0.042	1.01	1.000	0.040
	Shrub	2.35	0.185	0.073	1.18	1.000	0.038
	N-fix	1.66	1.000	0.046	1.68	0.493	0.047
	Herbaceous	**3.86**	**0.006**	**0.134**	**2.28**	**0.032**	**0.084**
	Bare	2.98	0.075	0.096	**2.26**	**0.008**	**0.075**
Seedling	Shrub	1.65	0.994	0.062	1.68	0.305	0.065
	N-fix	2.27	0.468	0.073	**2.53**	**0.027**	**0.083**
	Herbaceous	**3.43**	**0.032**	**0.146**	**2.40**	**0.024**	**0.112**
	Bare	**3.18**	**0.014**	**0.121**	**2.51**	**0.002**	**0.102**
Shrub	N-fix	1.56	1.000	0.044	1.23	1.000	0.035
	Herbaceous	2.10	0.135	0.078	1.91	0.172	0.071
	Bare	2.08	0.121	0.069	**2.08**	**0.026**	**0.069**
N-fix	Herbaceous	2.62	0.134	0.083	1.96	0.103	0.063
	Bare	1.66	1.000	0.049	2.01	0.058	0.059
Herbaceous	Bare	1.03	1.000	0.043	0.68	1.000	0.029

*The *p*-values are corrected by the false discovery rate [43].

Table S3. Marginal effects of terms in distance-based redundancy analyses for 16S and ITS along the variables soil total carbon (C), total nitrogen (N), resin-available nitrate (NO_3^-), resin-available ammonium (NH_4^+), resin-available phosphate (PO_4^{3-}), pH (1:2 $CaCl_2$), and water holding capacity (WHC). Edaphic measurements are from Dove et al. (6) using the same samples used for molecular analyses. Bolded values indicate significant terms (p < 0.05).

	Archaea/Bacteria (16S)		Fungi (ITS)	
	F-value	*p*-value	*F*-value	*p*-value
NO_3^-	1.499	0.113	0.629	0.950
NH_4^+	0.650	0.858	0.895	0.624
Total C	**2.912**	**0.004**	**2.498**	**< 0.001**
Total N	**2.210**	**0.018**	**2.117**	**0.002**
WHC	1.061	0.335	**1.936**	**0.004**
PO_4^{3-}	**2.013**	**0.028**	**1.568**	**0.034**
pH_{CaCl2}	**7.840**	**< 0.001**	**1.851**	**0.009**

Table S4. Mean (with SE) plot-scale soil properties of the chronosequence sites. Table is reproduced from Dove et al. [3].

	SOC[a] (%)	Soil N[b] (%)	pH[c]	WHC[d]	MAST[e]
4 y	7.81 (0.91)	0.38 (0.05)	5.57 (0.08)	0.81 (0.05)	12.6 (0.9)
13 y	4.36 (0.50)	0.17 (0.02)	4.83 (0.14)	0.83 (0.10)	12.7 (0.2)
25 y	6.42 (1.81)	0.29 (0.06)	4.73 (0.09)	0.83 (0.10)	11.3 (0.1)
>115 y	14.25 (2.69)	0.56 (0.12)	4.97 (0.17)	1.38 (0.18)	9.4 (0.7)

[a] Soil organic carbon.
[b] Nitrogen.
[c] 1:2 w/v 0.01 M $CaCl_2$.
[d] Water Holding capacity (kg kg^{-1}).
[e] Mean annual soil temperature (°C).

Table S5. Heatmap showing presence of functional potential (in yellow) in early- or late-successional metagenome assembled genomes. Table is presented in a separate spreadsheet.

Table S6. Description of metagenome assembled genomes. Table is presented in a separate spreadsheet.

SUPPLEMENTARY REFERENCES

1. Safford HD, van de Water KM. Using Fire Return Interval Departure (FRID) Analysis to Map Spatial and Temporal Changes in Fire Frequency on National Forest. 2014. USDA Forest Service Pacific Southwest Research Station.
2. Bohlman GN, North M, Safford HD. Shrub removal in reforested post-fire areas increases native plant species richness. *Forest Ecology and Management* 2016; **374**: 195–210.
3. Dove NC, Safford HD, Bohlman GN, Estes BL, Hart SC. High-severity wildfire leads to multi-decadal impacts on soil biogeochemistry in mixed-conifer forests. *Ecol Appl* 2020; e02072.

4. Dove NC. Soil Microbial Ecology of the Sierra Nevada: Predictions for a Warm and Fiery Future. 2019. Ph.D., University of California, Merced.

5. Parada AE, Needham DM, Fuhrman JA. Every base matters: assessing small subunit rRNA primers for marine microbiomes with mock communities, time series and global field samples. *Environmental Microbiology* 2016; **18**: 1403–1414.

6. Ihrmark K, Bödeker ITM, Cruz-Martinez K, Friberg H, Kubartova A, Schenck J, et al. New primers to amplify the fungal ITS2 region – evaluation by 454-sequencing of artificial and natural communities. *FEMS Microbiol Ecol* 2012; **82**: 666–677.

7. Edgar RC. Search and clustering orders of magnitude faster than BLAST. *Bioinformatics* 2010; **26**: 2460–2461.

8. Quast C, Pruesse E, Yilmaz P, Gerken J, Schweer T, Yarza P, et al. The SILVA ribosomal RNA gene database project: improved data processing and web-based tools. *Nucleic Acids Res* 2013; **41**: D590–D596.

9. Kõljalg U, Larsson K-H, Abarenkov K, Nilsson RH, Alexander IJ, Eberhardt U, et al. UNITE: a database providing web-based methods for the molecular identification of ectomycorrhizal fungi. *New Phytologist* 2005; **166**: 1063–1068.

10. Rodriguez-R LM, Konstantinidis KT. Nonpareil: a redundancy-based approach to assess the level of coverage in metagenomic datasets. *Bioinformatics* 2014; **30**: 629–635.

11. Bolger AM, Lohse M, Usadel B. Trimmomatic: a flexible trimmer for Illumina sequence data. *Bioinformatics* 2014; **30**: 2114–2120.

12. Menzel P, Ng KL, Krogh A. Fast and sensitive taxonomic classification for metagenomics with Kaiju. *Nature Communications* 2016; **7**: 11257.

13. Hyatt D, Chen G-L, LoCascio PF, Land ML, Larimer FW, Hauser LJ. Prodigal: prokaryotic gene recognition and translation initiation site identification. *BMC Bioinformatics* 2010; **11**: 119.
14. Cantarel BL, Coutinho PM, Rancurel C, Bernard T, Lombard V, Henrissat B. The Carbohydrate-Active EnZymes database (CAZy): an expert resource for Glycogenomics. *Nucleic Acids Res* 2009; **37**: D233–D238.
15. Lombard V, Golaconda Ramulu H, Drula E, Coutinho PM, Henrissat B. The carbohydrate-active enzymes database (CAZy) in 2013. *Nucleic Acids Res* 2014; **42**: D490–D495.
16. Buchfink B, Xie C, Huson DH. Fast and sensitive protein alignment using DIAMOND. *Nature Methods* 2015; **12**: 59–60.
17. Eddy SR. Accelerated Profile HMM Searches. *PLOS Computational Biology* 2011; **7**: e1002195.
18. Kanehisa M, Goto S. KEGG: Kyoto Encyclopedia of Genes and Genomes. *Nucleic Acids Res* 2000; **28**: 27–30.
19. Li D, Luo R, Liu C-M, Leung C-M, Ting H-F, Sadakane K, et al. MEGAHIT v1.0: A fast and scalable metagenome assembler driven by advanced methodologies and community practices. *Methods* 2016; **102**: 3–11.
20. Bushnell B. BBMap: A Fast, Accurate, Splice-Aware Aligner. 2014. Lawrence Berkeley National Lab. (LBNL), Berkeley, CA (United States).
21. Nurk S, Meleshko D, Korobeynikov A, Pevzner PA. metaSPAdes: a new versatile metagenomic assembler. *Genome Res* 2017; **27**: 824–834.

22. Wu Y-W, Simmons BA, Singer SW. MaxBin 2.0: an automated binning algorithm to recover genomes from multiple metagenomic datasets. *Bioinformatics* 2016; **32**: 605–607.

23. Kang DD, Froula J, Egan R, Wang Z. MetaBAT, an efficient tool for accurately reconstructing single genomes from complex microbial communities. *PeerJ* 2015; **3**: e1165.

24. Sieber CMK, Probst AJ, Sharrar A, Thomas BC, Hess M, Tringe SG, et al. Recovery of genomes from metagenomes via a dereplication, aggregation and scoring strategy. *Nature Microbiology* 2018; **3**: 836–843.

25. Parks DH, Imelfort M, Skennerton CT, Hugenholtz P, Tyson GW. CheckM: assessing the quality of microbial genomes recovered from isolates, single cells, and metagenomes. *Genome Res* 2015; gr.186072.114.

26. Xue Y, Jonassen I, Øvreås L, Taş N. Metagenome-assembled genome distribution and key functionality highlight importance of aerobic metabolism in Svalbard permafrost. *FEMS Microbiol Ecol* 2020; **96**.

27. Chaumeil P-A, Mussig AJ, Hugenholtz P, Parks DH. GTDB-Tk: a toolkit to classify genomes with the Genome Taxonomy Database. *Bioinformatics* 2019.

28. Bowers RM, Kyrpides NC, Stepanauskas R, Harmon-Smith M, Doud D, Reddy TBK, et al. Minimum information about a single amplified genome (MISAG) and a metagenome-assembled genome (MIMAG) of bacteria and archaea. *Nature Biotechnology* 2017; **35**: 725–731.

29. R Development Core Team. R: A language and environment for statistical computing. 2008. R Foundation for Statistical Computing, Vienna, Australia.

30. Lenth RV. Least-Squares Means: The R Package 'lsmeans'. *Journal of Statistical Software* 2016; **69**: 1–33.

31. Rohart F, Gautier B, Singh A, Cao K-AL. mixOmics: An R package for 'omics feature selection and multiple data integration. *PLOS Computational Biology* 2017; **13**: e1005752.

32. Pinheiro J, Bates D, DebRoy S, Sarkar D. nlme: Linear and Nonlinear Mixed Effects Models. 2017.

33. McMurdie PJ, Holmes S. phyloseq: An R Package for Reproducible Interactive Analysis and Graphics of Microbiome Census Data. *PLOS ONE* 2013; **8**: e61217.

34. Oksanen J, Blanchet FG, Kindt R, Legendre P, Simpson GL, Minchin PR, et al. vegan: Community Ecology Package. 2013.

35. Anderson MJ. A new method for non-parametric multivariate analysis of variance. *Austral Ecology* 2001; **26**: 32–46.

36. Bray JR, Curtis JT. An Ordination of the Upland Forest Communities of Southern Wisconsin. *Ecological Monographs* 1957; **27**: 325–349.

37. Nguyen NH, Song Z, Bates ST, Branco S, Tedersoo L, Menke J, et al. FUNGuild: An open annotation tool for parsing fungal community datasets by ecological guild. *Fungal Ecology* 2016; **20**: 241–248.

38. Stegen JC, Lin X, Fredrickson JK, Chen X, Kennedy DW, Murray CJ, et al. Quantifying community assembly processes and identifying features that impose them. *ISME J* 2013; **7**: 2069–2079.

39. Ning D, Yuan M, Wu L, Zhang Y, Guo X, Zhou X, et al. A quantitative framework reveals ecological drivers of grassland microbial community assembly in response to warming. *Nat Commun* 2020; **11**: 4717.
40. Fine PVA, Kembel SW. Phylogenetic community structure and phylogenetic turnover across space and edaphic gradients in western Amazonian tree communities. *Ecography* 2011; **34**: 552–565.
41. Chase JM, Kraft NJB, Smith KG, Vellend M, Inouye BD. Using null models to disentangle variation in community dissimilarity from variation in α-diversity. *Ecosphere* 2011; art24.
42. Stegen JC, Lin X, Fredrickson JK, Konopka AE. Estimating and mapping ecological processes influencing microbial community assembly. *Front Microbiol* 2015; **6**.
43. Benjamini Y, Hochberg Y. Controlling the False Discovery Rate: A Practical and Powerful Approach to Multiple Testing. *Journal of the Royal Statistical Society Series B (Methodological)* 1995; **57**: 289–300.
